# Thoracoscopic hiatoplasty in congenital diaphragmatic hernia is safe and less invasive: a prospective propensity-matched short-term study

**DOI:** 10.1007/s00383-025-06218-0

**Published:** 2025-10-21

**Authors:** Michaela Klinke, Richard Martel, Christel Weiß, Christoph Mohr, Thomas Schaible, Nina Dietze, Jana Hoffmann, Michael Boettcher, Julia Elrod

**Affiliations:** 1 Department of Pediatric Surgery, University Medical Center Mannheim, Heidelberg University, Theodor-Kutzer-Ufer 1-3, 68167 Mannheim, Germany; 2 Department of Medical Statistics and Biomathematics, Medical Faculty Mannheim, Heidelberg University, Theodor-Kutzer-Ufer 1-3, Mannheim, Germany; 3 Department of Neonatology, University Medical Center Mannheim, Heidelberg University, Theodor-Kutzer-Ufer 1-3, 68167 Mannheim, Germany

**Keywords:** CDH, Congenital diaphragmatic hernia, Hiatoplasty, Antireflux, Thoracoscopy, Minimal invasive surgery

## Abstract

**Background:**

Congenital diaphragmatic hernia (CDH) is a condition linked to neonatal morbidity, mortality, and gastroesophageal reflux. Traditional hiatoplasty via laparotomy or laparoscopy is complicated by abdominal adhesions from previous CDH repair. A thoracoscopic technique, avoiding abdominal preparation, was introduced to address these issues. This study evaluates its safety and efficacy in children with CDH.

**Methods:**

A prospective, propensity-matched study was conducted on pediatric patients undergoing thoracoscopic hiatoplasty (TH) or hiatoplasty via laparotomy for CDH at University Hospital Mannheim (2013–2024). Outcomes included operative time, ICU LOS and overall hospital stay (LOS), complication rates, and recovery, assessed via parent-reported questionnaires.

**Results:**

57 children underwent hiatoplasty via laparotomy, while 12 had TH. Propensity matching yielded 27 patients (laparotomy n = 19, TH n = 8). TH showed shorter operative time (71.5 vs 190.5 min; *p* = 0.0018), reduced ICU LOS (0.1 ± 0.3 vs 1.6 ± 4.0 days; *p* = 0.0002), and shorter overall LOS (2.4 ± 1.1 vs 13.2 ± 6.1 days; *p* < 0.0001). Complications were fewer (*p* = 0.0022), and dietary progression improved (*p* = 0.0181), with a trend toward earlier sports resumption (*p* = 0.0545). Follow-up duration was markedly shorter in the TH group (median 109 days [IQR 70–151] vs. 1465 days [IQR 924–2390], *p* = 0.0041).

**Conclusion:**

TH is a safe, effective antireflux therapy for CDH, offering better recovery and fewer complications. Interpretation is limited by the shorter follow-up in the thoracoscopic group. Long-term randomized trials are needed to confirm these results.

## Introduction

Congenital diaphragmatic hernia (CDH) is a severe congenital malformation characterized by the herniation of abdominal organs into the thoracic cavity due to a diaphragmatic defect. This condition leads to an abnormal lung development, resulting in lung hypoplasia, cardiac dysfunction and pulmonary hypertension [1–3]. CDH is associated with various complications and comorbidities such as respiratory distress, feeding challenges, growth retardation, and neurodevelopmental delays, all of which increase morbidity and mortality rates in affected infants [1, 4].

A common issue in children with CDH is gastroesophageal reflux (GER), primarily due to anatomical disruptions caused by the hernia and its surgical correction [4–7]. The repair of the diaphragmatic defect can create a gaping hiatus, increasing susceptibility to severe reflux [6, 8]. This exacerbates respiratory problems, impairs nutritional intake, and can lead to esophagitis and other gastrointestinal issues [1, 4, 9]. The symptoms of GER in children are diverse and age-dependent. Older children often report typical heartburn symptoms, such as retrosternal and epigastric pain, while infants and younger children commonly present with regurgitation. Additionally, failure to thrive, pulmonary symptoms such as coughing, increased incidence of upper respiratory tract infections, and apparent life-threatening events can occur. Complications like esophagitis, stricture formation, and ulcers may result in pain, dysphagia, and hemorrhage [10]. Proper management of the gaping hiatus is crucial for alleviating these symptoms and improving the quality of life for children with CDH. In the past, there have been repeated attempts to improve the outcome of patients with CDH by routinely performing anti-reflux procedures at the time of initial congenital diaphragmatic hernia repair. However, this approach has largely been abandoned as no benefit was demonstrated [6, 11–13].

Traditionally, hiatoplasty is performed via laparotomy or laparoscopy, both of which require extensive adhesiolysis and carry a risk of bowel and organ damage [14]. These approaches are often followed by transient postoperative ileus, slow dietary progression, and delayed recovery [15]. Recently, a minimally invasive thoracoscopic method (thoracoscopic hiatoplasty, TH) has been introduced at our institution to treat a gaping hiatus in children with CDH-related reflux symptoms. The primary hypothesis is that this new approach will promote faster recovery, shorten hospital stays, and reduce perioperative complications, while being equally effective in symptom relief as the gaping hiatus may be the most important reason for GERD in these patients. This prospective study aims to systematically evaluate and compare the outcomes of the TH with the traditional open approach to determine the effectiveness of the TH in advancing evidence-based therapeutic practices.

## Materials and methods

### Study group

A prospective observational study using a propensity score matched analysis was conducted on data from paediatric patients who underwent surgical repair for recurrent CDH at the Department for Pediatric Surgery at the University Hospital Mannheim, University Heidelberg between 01/2010 and 04/2024. Patients were categorised into two cohorts based on the surgical technique employed for the repair: laparotomy and TH. Hiatoplasty was performed in CDH patients with clinically relevant GER symptoms or complications, as described below. All such patients were considered; no further exclusion criteria were applied.

Prospective data collection took place during the initial hospital stay, during the routine follow ups at the age of 6 months, 12 months, 24 months, 4 years, 6 years, 10 years, 14 years and 17 years and at all additional presentations to the hospital. The following baseline characteristics were assessed: gender, gestational age, birth weight, size and laterality of the diaphragmatic defect, extracorporeal membrane oxygenation (ECMO) therapy, initial surgical approach (laparotomy vs. thoracoscopy) and use of a patch in the primary CDH repair. Furthermore, parameters regarding hiatoplasty such as age at time of hiatoplasty, surgical approach (laparotomy vs. TH), intraoperative complications, operative time, requirement for ICU admission, ICU length of stay (ICU LOS), overall hospital length of stay (LOS), and postoperative complications, such as necessity for dilation of the hiatus due to dysphagia or redo surgery due to recurrent gastroesophageal complaints were evaluated.

### Ethics

This study was performed in accordance with the Helsinki Declaration as revised in 2013 and was approved by the competent ethical committee (study ID 2022–626).

Informed consent was obtained from all subjects and/or their legal guardian(s).

### Surgical indications and preoperative workup

Hiatoplasty was generally performed in patients with significant clinical symptoms suggestive of GERD or its complications. Typical indications included failure to thrive, recurrent or persistent reflux symptoms, and pulmonary issues such as recurrent aspiration or respiratory distress suspected to be related to GERD. Preoperative diagnostics typically included a combination of upper gastrointestinal endoscopy with mostly, but not always histopathological biopsy, as well as 24-h impedance and pH-metry, to confirm the presence and severity of reflux. In some cases, the decision for surgical intervention was supported by imaging studies, such as contrast radiography, or was based on a combination of clinical judgment and diagnostic findings, particularly when a gaping hiatus was suspected.

### Surgical techniques

The surgical procedures for hiatoplasty evaluated in this study included both TH and laparotomy. In the laparotomy cohort, a median incision was performed, followed by complete adhesiolysis to fully expose the hiatus. For the TH, patients were positioned in a modified prone position with elevation of the right side, and three trocars were placed intercostally along the anterior to mid-axillary line. Simultaneous endoscopy was utilized to accurately identify the cardia and hiatus. In both approaches, the crus sinistrum and crus dextrum of the pars lumbalis diaphragmatis were exposed. Posterior hiatoplasty, and anterior if necessary, were performed using braided single-knot sutures. During the TH procedure, the success of the intervention was immediately assessed with simultaneous endoscopy. The intraoperative criteria for effectiveness of the procedure were: Smooth passage of the endoscope through the level of the hiatoplasty while spontaneous opening of the cardia was eliminated/repaired. In certain cases, additional procedures, such as the repair of a recurrent hernia, were also carried out (see Table 1).

**Table 1:** Basic patient characteristics of all patients with hiatoplasty via laparotomy or TH. A total of 12 patients underwent TH, whereas 57 underwent hiatoplasty via laparotomy. Differences between the two groups were calculated using Fisher’s Exact Test, Two Sample t-test and Cochran-Armitage test for trend. n/(%); Mean (SD)

Characteristics	Laparotomy n = 57	TH n = 12	*P*-value
Gender			0.1268
f	28 (49%)	3 (25%)	
m	29 (51%)	9 (75%)	
Week of gestation	36.9 ± 2.4	38.8 ± 1.3	**0.0014**
Missing	4	0	
Birth weight [g]	2678.8 ± 560.2	3108.2 ± 510.1	**0.0341**
Missing	5	1	
Defect size			1.0000
B	8 (16%)	1 (10%)	
C	27 (55%)	6 (60%)	
D	14 (29%)	3 (30%)	
Missing	8	2	
Defect location			1.0000
Left	56 (98%)	12 (100%)	
Right	1 (2%)	0	
ECMO treatment	33 (58%)	5 (42%)	0.3043
Surgical access of closure of CDH			0.4637
Laparotomy	51 (96%)	11 (91%)	
Thoracoscopy	2 (3%)	1 (8%)	
Missing	4	0	
Type of closure of the CDH			0.0760
Patch	56 (98%)	10 (83%)	
Age at time of hiatoplasty [days]	1043.0 ± 1077.2 median: 669 IQR: [292, 1473]	1991.0 ± 1239.3 median: 1716 IQR: [931, 3003.5]	**0.0041**
Cases in which additional procedures were conducted during the same surgery	37 (65%)	4 (33%)	0.0563
Follow-up duration after hiatoplasty [days]	1736.4 ± 1115.3 Median: 1465 IQR: [924, 2390]	109.4 ± 61.6 Median: 109 IQR: [70, 151]	** < 0.0001**

*CI* confidence interval, *EMCO* extracorporeal membrane oxygenation, *m* male, *f* female, *TH* thoracoscopic hiatoplasty

### Questionnaires

Parent-reported assessment of a number of parameters addressing the postoperative phase and the period after discharge was performed, using a questionnaire specially designed for this study. The questionnaire was specifically designed for this study, piloted internally with a small group of caregivers, but not formally validated. It was administered to the legal guardians of the young patients, who were contacted by telephone and responded via the tool LimeSurvey [16]. Families were contacted up to three times in an attempt to reach as many as possible; participation in the survey was voluntary. For the complete questionnaire, see Tables 2 and 3.

**Table 2:** Operation and outcome parameters of all patients with hiatoplasty via laparotomy or TH. Differences between the two groups were calculated using Fisher`s Exact Test, Two Sample t-test and Cochran-Armitage Trend-Test. *P*-values ≤ 0.05 were considered significant

Characteristics	Laparotomy n = 57	TH n = 12	*P* value
Duration of surgery, all cases [min]	Median: 220.0 IQR: [207,243]	Median: 121.5 IQR: [60,168]	**0.0001**
Duration of surgery, only cases **without** additional procedure NEU [min]	n = 20 Median: 190.5 IQR: [169, 225]	n = 8 Median: 71.5 IQR: [58, 124]	**0.0018**
Stay in ICU [%]	35 (76%)	1 (8%)	** < 0.0001**
Missing	11	0	
Stay in ICU [days]	Median: 1 Range: [0,6] 1.6 ± 4.0	Median: 0 Range: [0,0] 0.1 ± 0.3	**0.0002**
Missing	11	0	
Stay in hospital total [days]	Median: 12 IQR: [6, 10] 13.2 ± 6.1	Median: 2 IQR: [1, 4] 2.4 ± 1.1	** < 0.0001**
Missing	13	1	
Complications			
Including minor	39 (68%)	2 (17%)	**0.0022**
Only redo-surgery / oesophageal dilation	5 (9%)	1 (8%)	1.0000

*ECMO* extracorporeal membrane oxygenation, *ICU* intensive care unit, *LOS* length of stay, *TH* thoracoscopic hiatoplasty

**Table 3:** Questionnaire regarding hospital stay and early postoperative course. A total of 11 patients who underwent TH, and 32 who underwent hiatoplasty via laparotomy agreed to complete the questionnaire. Differences between the two groups were calculated using Cochran–Armitage test for trend. *P*-values < 0.05 were considered significant

	Laparotomy n = 32	TH n = 11	*p*-value
How long did your child experience **vomiting** after the surgery?			0.4110
Not at all	24 (75%)	9 (82%)	
Maximum of 1 day	0	1 (9%)	
Several days	4 (12.5%)	1 (9%)	
Several weeks or persisting	4 (12.5%)	0	
How long did your child experience **postoperative pain**?			0.6246
Not at all	0	1 (9%)	
Maximum of 1 day	8 (25%)	3 (27%)	
Several days	19 (59%)	5 (45%)	
Several weeks or persisting	5 (16%)	2 (18%)	
Did your child have difficulty swallowing when **drinking** after the surgery?			0.8791
Not at all	23 (72%)	7 (64%)	
Maximum of 1 day	2 (6%)	2 (18%)	
Several days	3 (9%)	0	
Several weeks or persisting	4 (12.5%)	2 (18%)	
Did your child have difficulty swallowing when **eating** after the surgery?			0.8872
Not at all	23 (72%)	7 (64%)	
Maximum of 1 day	1 (3%)	2 (18%)	
Several days	4 (12.5%)	0	
Several weeks or persisting	4 (12.5%)	2 (18%)	
How long did the **complete dietary progression** take?			**0.0181**
Just one day	7 (22%)	8 (73%)	
Several days	13 (41%)	2 (18%)	
Several weeks	4 (12.5%)	0	
Several months or persisting	8 (25%)	1 (9%)	
How long did it take for your child to regain full **mobility** after the surgery?			0.4685
Just one day	7 (22%)	5 (45%)	
Several days	20 (62.5%)	4 (36%)	
Several weeks	4 (12.5%)	2 (18%)	
Several months or persisting	1 (3%)	0	

*TH* thoracoscopic hiatoplasty

### Outcomes

The primary outcomes of this study were the ICU LOS, overall hospital LOS, duration of surgery and rate of complications. Secondary outcomes were the results from the questionnaires.

### Statistical analysis

Statistical analysis was performed R (Version 4.3.2; R Core Team 2021), RStudio (Version 2023.12.0) and SAS (SAS Institute Inc., North Carolina, Release 9.4, 2023). Continuous variables such as week of gestation, birth weight, age at recurrence, operating time of CDH repair, ICU LOS and overall hospital LOS were expressed as mean and standard deviation. Binary or categorical variables, including sex, ECMO therapy, size and laterality of the initial diaphragmatic defect, operation technique, as well as the performance of additional procedures during hiatoplasty surgery and the occurrence of complication, were presented as absolute and relative frequencies. Regarding the questionnaires, the answers were coded into numbers. Cochran–Armitage tests for trends were applied to compare the results between hiatoplasty via laparotomy and TH groups.

To assess possible differences between the two treatment groups, *p*-values were calculated. Continuous variables were analysed using Wilcoxon 2 sample tests, ordinal variables were assessed with the exact Cochran-Armitage test for trends, and binary and categorical variables were examined using the chi-square test or Fisher’s exact test. To control for known confounding factors (gender, gestational age, requirement of ECMO therapy, age at time of recurrence operation), propensity score matching was performed. Consecutively, TH patients were matched with up to three laparotomy patients. Analysis regarding duration or surgery was conducted including all cases and separately only for cases without additional procedures. *P*-values < 0.05 were considered statistically significant.

## Results

### Descriptive analysis: comparison of baseline characteristics

Surgical intervention for hiatoplasty was performed on a total of 69 children at the department during the period from 2010 to 2024. If the surgery had to be repeated again during the years of follow-up, only the first procedure was included into statistics. Of these, 57 (82%) hiatoplasties were managed via laparotomy, and 12 (17%) via TH. For details see Table 1.

When comparing the baseline characteristics of both patient groups there are significant differences concerning the week of gestation (laparotomy = 36.9 ± 2.4 weeks, TH = 38.8 ± 1.3 weeks, *p* = 0.0014) and the birth weight (laparotomy = 2678.8 ± 560.2, TH = 3108.2 ± 510.1 g; *p* = 0.0341). Another significant difference was the age at time of hiatoplasty (laparotomy: median: 669 days, IQR: [292, 1473], TH: median: 1716, IQR: [931, 3003.5], *p* = 0.0041). Importantly, since TH was only recently introduced to our clinic, whereas hiatoplasty via laparotomy date back to 2010, follow-up duration differed significantly between the two groups (laparotomy: Median: 1465, IQR: [924, 2390], TH: median: 109, IQR: [70, 151]). All other parameters, most importantly defect size and the condition of requirement of neonatal ECMO therapy did not differ between the two groups.

### Comparison of intraoperative and outcome parameters

**Fig. 1:**
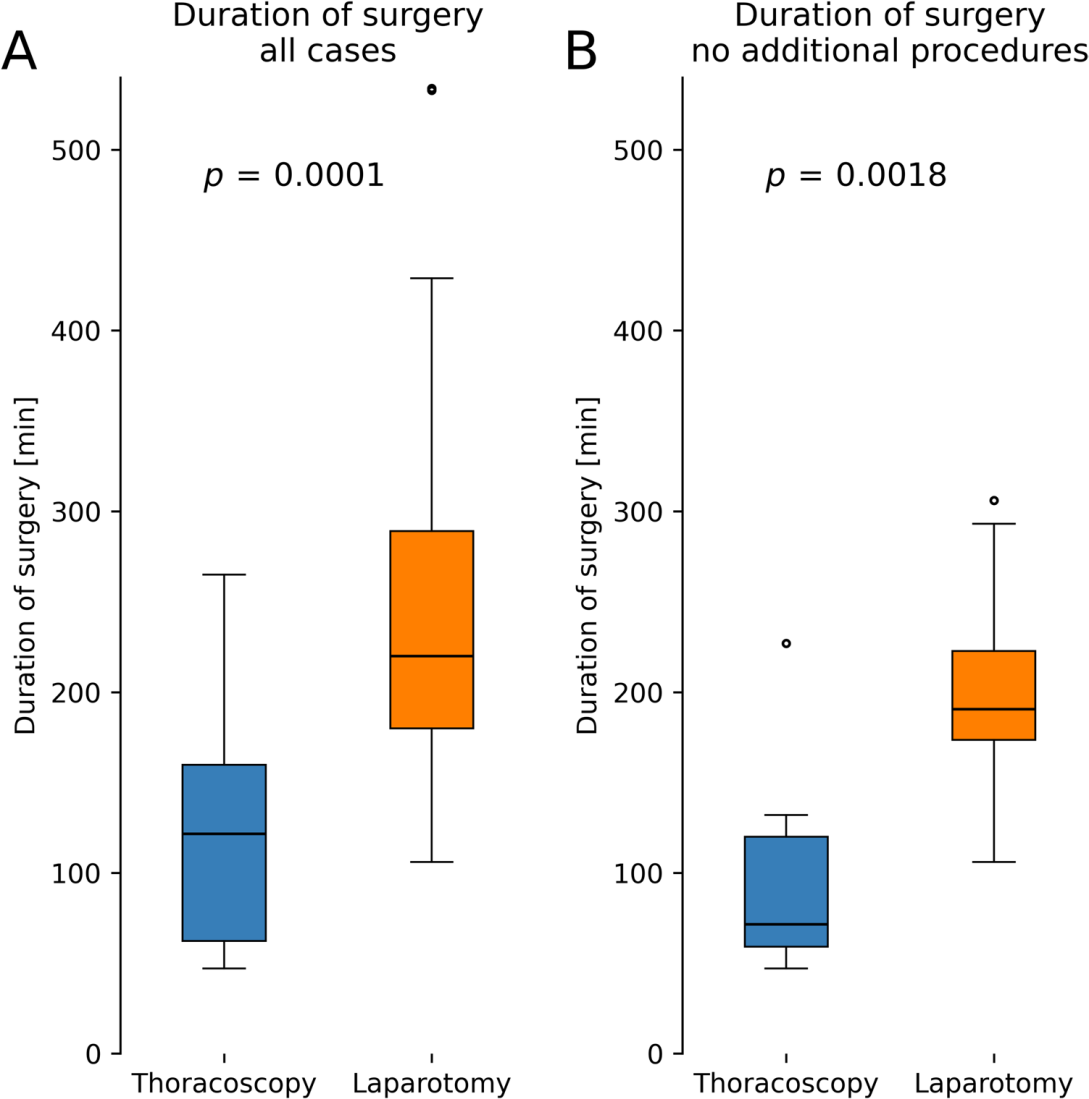
The duration of surgery is significantly shorter in patients with TH, compared to hiatoplasty via laparotomy. **A** Figure showing all cases of hiatoplasty via laparotomy (n = 57) and all TH approaches (n = 12). In the laparotomy group median duration of surgery was 220.0 min, whereas in the TH group, it was only 121.5 min (*p* = 0.0001). **B** Figure showing only surgeries without additional procedures, such as repair of recurrence: In the laparotomy group median duration of surgery was 190.5 min, whereas in the TH group, it was 71.5 min (*p* = 0.0018). Whiskers represent 2.5 to 97.5 of all data. Abbreviations: TH thoracoscopic hiatoplasty

**Fig. 2:**
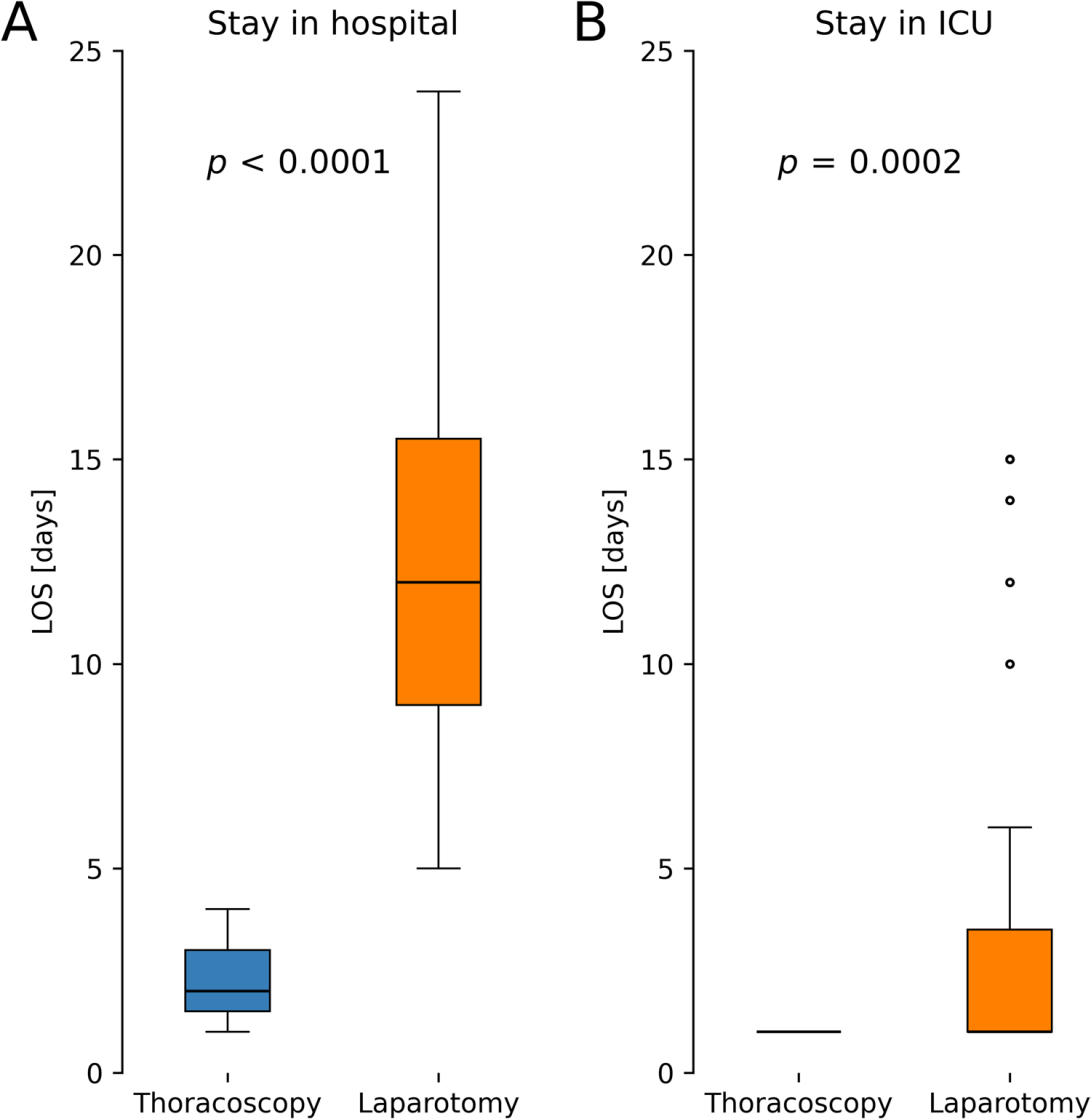
Overall LOS and ICU LOS only is significantly shorter in patients with TH, compared to hiatoplasty via laparotomy. In the laparotomy group (n = 57) mean overall LOS was 13.2 ± 6.1 days, whereas in TH group (n = 12) it was 2.4 ± 1.1 days (< 0.0001). Of the laparotomy group, 76% of all children required ICU admission postoperatively, whereas only 1 child (8%) had to stay there in the TH group. This results in a significantly lower overall LOS in the TH group (laparotomy: 1.6 ± 4.0 days; TH: 0.1 ± 0.3 days, *p* = 0.0002). Whiskers represent 2.5 to 97.5 of all data. Abbreviations: ECMO extracorporeal membrane oxygenation, LOS length of stay, ICU intensive care unit, TH thoracoscopic hiatoplasty

Intraoperative parameters and postoperative outcomes were compared between the laparotomy and the TH group (Table 2). The findings indicate that the duration of the surgical procedure was significantly reduced in the TH group: When taking into account all cases of hiatoplasty, median time of surgery was 220.0 min in the open group and only 121.5 min in the TH group (*p* = 0.0001), see Fig. 1 and Table 3. Subgroup analysis was conducted only for surgeries in which no additional procedures, such as repair of recurrent CDH were performed. Here median duration of surgery was 190.5 min in the open group vs. 71.5 min in the TH group (*p* = 0.0018). Favorable outcome of the TH group was also observed regarding the requirement for ICU admission, occurring in only one case (8%), whereas it was needed in the majority of all cases (76%) in the open group (*p* < 0.0001). Moreover, children in the open group required a longer mean ICU stay (mean: 1.6 ± 4.0 days) than the TH group (mean: 0.1 ± 0.3 days, *p* = 0.0002), and overall LOS was also significantly longer (mean: 13.2 ± 6.1) than in the minimal invasive group (mean: 2.4 ± 1.1) (*p* < 0.0001), see Fig. 2.

When counting all complications, including e.g. intraoperative immediate injury of the small intestine, that necessitated individual stitches, which however resolved without sequelae, or obstruction requiring oesophageal dilation or redo surgery, these were observed in 39 (68%) of patients treated with hiatoplasty via laparotomy, and in 2 (17%) patients treated with TH, (*p* = 0.0022). The most common minor complication was serosal injury. Conversely, redo surgery or dilation was necessary in n = 5 (9%) cases in the laparotomy group, and in n = 1 (8%) case in the TH group. Yet, statistical comparison is not feasible due to small number and due to the significant shorter follow-up duration in the TH group, which can be a confounding factor, since re-do surgeries might occur up to years later. For details see Table 4.

**Table 4:** Questionnaire regarding the time after discharge home. A total of 11 patients who underwent TH, and 32 who underwent hiatoplasty via laparotomy agreed to complete the questionnaire. Differences between the two groups were calculated using Cochran–Armitage test for trend. *P*-values < 0.05 were considered significant

	Laparotomy n = 32	TH n = 11	*p*-value
How long did it take for your child to fully **recover** from the surgery?			0.1243
Just one day	2 (6%)	5 (45%)	
Several days	16 (50%)	2 (18%)	
Several weeks	10 (31%)	3 (27%)	
Several months or persisting	4 (12.5%)	1 (9%)	
How long did it take for your child **to return to daycare/kindergarten/school**?			0.3346
Just one day	0	4 (36%)	
Several days	14 (44%)	2 (18%)	
Several weeks	13 (41%)	2 (18%)	
Several months or persisting	5 (16%)	3 (27%)	
How long did it take for your child to resume **sports** activities?			**0.0545**
Just one day	0	4 (36%)	
Several days	14 (44%)	3 (27%)	
Several weeks	14 (44%)	3 (27%)	
Several months or persisting	4 (12.5%)	1 (9%)	

*TH* thoracoscopic hiatoplasty

### Evaluation of questionnaires

A total of 32 patients from the laparotomy group answered the online questionnaires, in the TH group, 11 patients were reached by telephone and fully answered the survey. The TH group showed favourable results in comparison to the open group regarding time to complete dietary progression (*p* = 0.0181). Moreover, children were able to resume sports earlier. This result however slightly failed to reach statistical significance (*p* = 0.0545).

### Propensity scoring

Propensity scoring was performed to correct for the possible confounding factors gender, gestational age, requirement of ECMO therapy and age at time of hiatoplasty. For the purpose of propensity matching, 1–3 patients of the laparotomy group were matched to every patient of the TH group, resulting in 19 patients in the laparotomy group and 8 patients in the TH group. Now both groups included subjects with similar distribution of gender (*p* = 0.2357), week of gestation (*p* = 0.9787), requirement for ECMO treatment (*p* = 1.0000) and age at time of repair of hiatoplasty (*p* = 0.1442).

Comparison of the matched groups showed no difference regarding the percentage of surgeries in which additional procedures, such as repair of recurrence were performed. When taking into account all surgeries, duration of surgery was significantly shorter in the TH group (laparotomy: Median: 231 IQR: [192, 292] vs TH Median: 1739.5 IQR: [832, 3003]). Including only cases without additional procedures to the analysis regarding duration of surgery, reduced the number to n = 8 in the laparotomy group and n = 4 in the TH group, the latter still showing a somewhat favorable outcome, however without reaching a level of significance (laparotomy: Median: 204.5 IQR: [185, 261.5], TH: Median: 71.5, IQR: [55, 153.5]; *p* = 0.0745). Moreover, children in the TH group had a lower likelihood of requirement of ICU treatment (laparotomy: n = 11 (61%) vs TH: n = 0 (0%); *p* = 0.0078), and if required also a shorter stay on the ICU (laparotomy: Median: 1 day, IQR: [0, 1] vs. TH: Median: 0 days, IQR: [0, 0]; *p* = 0.0103). Likewise overall hospital stay was shorter in the minimal invasive group (laparotomy: Median: 12, IQR: [8, 14] vs TH: Median: 3 days, IQR: [2, 4]; *p* = 0.0002).

Furthermore, the rate of complications was favorable in the TH group including all complications, of which most were minor, such as serosal injury during surgery (laparotomy: n = 15 (79%) vs TH: n = 2 (25%), *p* = 0.0248), however regarding only redo-surgeries or the need for complications, there were no differences. Importantly however again comparability is limited due to the shorter follow-up period for the laparotomy patients, recognizing that redo surgeries for recurrent GER often become necessary much later. See the results of propensity scored, matched baseline characteristics and outcome parameters in Table 5.

**Table 5:** Propensity scored, matched baseline characteristics and outcome parameters in patients with hiatoplasty via laparotomy or TH. After propensity score matching, most baseline characteristics were similar in both groups. Differences between the two groups were calculated using Fisher`s Exact Test, Chi-Square Test and Welch Two Sample t-test

	Laparotomy n = 19	TH n = 8	*p*-value
*Baseline characteristics*			
Gender			0.2357
f	10 (52.6%)	2 (25.0%)	
m	9 (47.4%)	6 (75.0%)	
Week of gestation	38.3 ± 0.9	38.1 ± 0.8	0.9787
ECMO treatment	12 (63.2%)	5 (62.5%)	1.0000
Age at time of hiatoplasty [days]	Median: 827 IQR: [425, 1756]	Median: 1739.5 IQR: [832, 3003]	0.1442
*Outcome parameter*			
% of surgeries with additional procedures	11 (57.9%)	4 (50.0%)	1.0000
Duration of surgery, all cases [min]	Median: 231 IQR: [192, 292]	Median: 142 IQR: [71.5, 197.5]	**0.0100**
Duration of surgery, only without additional procedures [min]	Median: 204.5 IQR: [185, 261.5] (n = 8)	Median: 71.5 IQR: [55, 153.5] (n = 4)	0.0745
Stay in ICU [incidence]	11 (61.1%)	0 (0.00%)	**0.0078**
Missing	2	1	
Stay in ICU [days]	Median: 1 IQR: [0, 1]	Median: 0 IQR: [0, 0]	**0.0103**
Missing	2	0	
Stay in hospital total [days]	Median: 12 IQR: [8, 14]	Median: 3 IQR: [2, 4]	**0.0002**
Missing	2	1	
Complications, including minor	15 (79.0%)	2 (25.0%)	**0.0248**
Complications, only re-operation and dilation	3 (15.8%)	1 (12.5%)	**1.0000**

*ECMO* extracorporeal membrane oxygenation, *m* male, *f* female, *ICU* intensive care unit, *LOS* length of stay, *TH* thoracoscopic hiatoplasty

## Discussion

The objective of this study was to evaluate the safety and efficacy of TH in pediatric patients with congenital diaphragmatic hernia (CDH) compared to traditional hiatoplasty via laparotomy.

This preliminary analysis identified several advantages of TH over the laparotomy approach. Specifically, the median duration of surgery was significantly shorter in the TH group compared to the laparotomy group. Furthermore, TH was associated with a significantly reduced need for intensive care, and the overall length of hospital stay was substantially shorter. Intraoperative complications were vastly lower in TH compared to the laparotomy group. Furthermore, the TH showed superior outcomes in some of the aspects regarding the peri and postoperative period, according to parent reported outcomes.

The significant reduction in operative duration observed in the TH group can primarily be attributed to the avoidance of extensive adhesiolysis typically required in open procedures [17]. Extensive adhesiolysis is often necessary during laparotomy to expose the hiatus in children with CDH due to their inherent history of previous surgeries [7]. This essential step not only prolongs surgery but might also increase the risk of bowel injury and bleeding from organs, potentially extending recovery time[18]. In contrast, TH, which addresses the hiatus from the thoracic side, avoids the need for a large abdominal incision and, in particular, extensive peritoneal adhesiolysis. The tissue is only minimally manipulated, reducing the risk of intraoperative complications and also potentially leads to less postoperative (sub-)ileus [19], possibly leading to a faster recovery, including shorter time to complete dietary progression observed in the minimally invasive group. This reduction in tissue injury and operative duration likely explains the decreased need for ICU treatment postoperatively observed in this study. The reduced ICU dependency not only conserves healthcare resources but also minimizes the psychological impact of prolonged hospital stays on pediatric patients and their families, which is particularly important in chronically ill patients, such as those with severe forms of CDH.

Moreover, avoiding the abdominal incision is very likely associated with an earlier ability to return to normal activities, including sports and complete dietary progression, as assessed in the questionnaires. The latter was significant, highlighting the benefits of TH in promoting quicker overall recovery. These factors might collectively contribute to the shorter surgical duration and faster recovery in the TH group. Importantly, avoiding the abdominal incision and extensive adhesiolysis might also reduce the risk of future adhesions, which is crucial in a patient cohort where future interventions and surgeries are often necessary. However, this effect cannot be systematically studied in the present cohort due to the short follow-up period, as TH was only introduced in our center in autumn 2023.

While minor complications, such as serosal injury, were more common in the laparotomy group, the TH group experienced significantly less of such complications. Importantly, there was no significant difference in the necessity for reoperations or the need for dilation between the two groups, suggesting that TH may be as effective as hiatoplasty via laparotomy in managing GER. However, this finding must be interpreted cautiously, given the short follow-up duration in the TH group, which limits the ability to assess the long-term need to repeat hiatoplasties in the event of GER recurrence. Moreover, long-term studies are required to determine whether GER related symptoms improve equally in both groups. Systematic device-assisted investigations, including impedance and pH-metry, and, if necessary, esophagogastroduodenoscopy, are needed to thoroughly assess the benefits of this new technique [20].

Given the high incidence of reflux in patients with CDH—affecting up to 50%—and its associated long-term complications, such as oral aversion, failure to thrive, the need for positional adjustments, thickening agents, acid suppressants, or even long-term tube feeding [6, 7, 21]— adopting a minimally invasive surgical approach is imperative. This approach could significantly reduce the procedural burden and complication rates, enhancing recovery and overall outcomes for these patients.

In 1996, Sartotelli and Rothenberg reported a case study on the thoracoscopic repair of paraesophageal hiatal hernias in children post-fundoplication [22]. Similar to our CDH cohort, they opted for a thoracic approach to minimize risks such as bowel, liver, or esophageal injuries and to reduce prolonged operative times due to previous surgeries. They used contrast swallow studies and endoscopy for preparation, while we relied primarily on esophagogastroduodenoscopy with 24-h impedance-pH monitoring. Rothenberg’s approach involved a modified prone position from the left with lung mobilization, whereas we used a right-sided approach without needing lung mobilization or single-lung ventilation. Both procedures involved simultaneous endoscopy for identifying structures and verifying success. Although Rothenberg used four trocars while we used three, the surgical steps for hiatus preparation, hernia reduction, and crural repair were identical. Rothenberg’s team routinely placed a thoracic drain, which was rarely necessary in the current study. Their reported average operating time was 80 min, comparable to our median of 71 min. Follow-up in their study involved routine contrast studies, while ours was primarily clinical. Both, their case report and our study, suggest that TH is a safe and effective option for patients with prior abdominal surgeries.

Despite its benefits, TH remains a technically demanding procedure, requiring significant expertise and a steep learning curve. The delicate nature of the surgery, which involves working in close proximity to the heart, aorta, vena cava and other critical structures, necessitates precision and skill. Therefore, this procedure should be reserved for experienced surgeons in high-volume centers where surgical teams are well-versed in thoracoscopic techniques. In addition, it also requires an experienced endoscopist, not least because of the positioning of the patient and the resulting more difficult endoscopy conditions. The complexity of the procedure underscores the importance of adequate training and experience to ensure patient safety and optimal outcomes.

The present study has several limitations. The sample size was relatively small, especially for the TH group, and the follow-up duration was shorter in this group compared to the open group. These factors may influence the observed outcomes, limits interpretation of reoperation and might obscure long-term outcomes. Additionally, the non-randomized nature of the study introduces potential selection bias, which could affect the results. However, a significant bias is unlikely since, after the introduction of TH in the fall of 2023, this new procedure became the standard practice. Moreover, the indication for hiatoplasty was not entirely uniform, as clinical decisions were influenced by individual patient presentations and diagnostic findings, which may introduce variability in the study cohort. Another weakness is the subjective nature of the results from the retrospective surveys regarding postoperative recovery and time at home. Ideally, this data should be collected prospectively and as objectively as possible. It should also be noted that a recall effect probably introduces bias, due to the differences in follow-up duration. Furthermore, blinding of the patients and caregivers is not feasible, possibly skewing the results. Objective assessment of outcomes regarding GER symptoms should also be performed, including device-assisted measurements such as impedance and pH-metry, or even esophagogastroduodenoscopy including biopsy sampling. Future studies should include larger, randomized controlled trials with longer follow-up periods to validate these findings and assess the long-term efficacy and safety of TH in pediatric CDH patients.

## Conclusion

This preliminary analysis suggests that TH is a safe and effective antireflux therapy for children with congenital diaphragmatic hernia. It is associated with shorter operative times, reduced ICU dependency, decreased overall hospital stays, and lower complication rates compared to traditional hiatoplasty via laparotomy. However, given the technical demands and steep learning curve of this procedure, it should be performed exclusively by experienced surgeons in high-volume centers. Sufficiently powered, randomized controlled trials are necessary to confirm these preliminary findings and to validate the long-term outcomes.

## Data Availability

The data that support the findings of this study are available from the corresponding author upon reasonable request and with permission of our ethics committee.

## Abbreviations

CDH: Congenital diaphragmatic hernia
CT: Computed tomography
ECMO: Extracorporeal membrane oxygenation
GER: Gastroesophageal reflux
ICU: Intensive care unit
IQR: Interquartile range
LOS: Length of stay
MIS: Minimally invasive surgery
MRI: Magnetic resonance imaging
SD: Standard deviation
TH: Thoracoscopic hiatoplasty
